# Rediscovery of poly(ethylene glycol)s as a cryoprotectant for mesenchymal stem cells

**DOI:** 10.1186/s40824-023-00356-z

**Published:** 2023-02-20

**Authors:** Madhumita Patel, Jin Kyung Park, Byeongmoon Jeong

**Affiliations:** Department of Chemistry and Nanoscience, Ewha Womans University, 52 Ewhayeodae-Gil, Seodaemun-Gu, Seoul, 03760 Korea

**Keywords:** Cryoprotection, Poly(ethylene glycol), Molecular weight, Permeability, Ice recrystallization

## Abstract

**Background:**

A medium containing dimethyl sulfoxide (DMSO) (10% v/v) is most widely used for cell cryopreservation at –196 °C. However, residual DMSO consistently raises concerns because of its toxicity; thus, its complete removal process is required.

**Method:**

As biocompatible polymers approved by the Food and Drug Administration for various biomedical applications for humans, poly(ethylene glycol)s (PEGs) with various molecular weights (400, 600, 1 K, 1.5 K, 5 K, 10 K, and 20 K Da) were studied as a cryoprotectant of mesenchymal stem cells (MSCs). Considering the cell permeability difference of PEGs depending on their molecular weight, the cells were preincubated for 0 h (no incubation), 2 h, and 4 h at 37 °C in the presence of PEGs at 10 wt.% before cryopreservation at –196 °C for 7 days. Then, cell recovery was assayed.

**Results:**

We found that low molecular weight PEGs (400 and 600 Da) exhibit excellent cryoprotecting properties by 2 h preincubation, whereas intermediate molecular weight PEGs (1 K, 1.5 K, and 5 K Da) exhibit their cryoprotecting properties without preincubation. High molecular weight PEGs (10 K and 20 K Da) were ineffective as cryoprotectants for MSCs. Studies on ice recrystallization inhibition (IRI), ice nucleation inhibition (INI), membrane stabilization, and intracellular transport of PEGs suggest that low molecular weight PEGs (400 and 600 Da) exhibit excellent intracellular transport properties, and thus the internalized PEGs during preincubation contribute to the cryoprotection. Intermediate molecular weight PEGs (1 K, 1.5 K, and 5 K Da) worked by extracellular PEGs through IRI, INI, as well as partly internalized PEGs. High molecular weight PEGs (10 K and 20 K Da) killed the cells during preincubation and were ineffective as cryoprotectants.

**Conclusions:**

PEGs can be used as cryoprotectants. However, the detailed procedures, including preincubation, should consider the effect of the molecular weight of PEGs. The recovered cells well proliferated and underwent osteo/chondro/adipogenic differentiation similar to the MSCs recovered from the traditional DMSO 10% system.

**Graphical Abstract:**

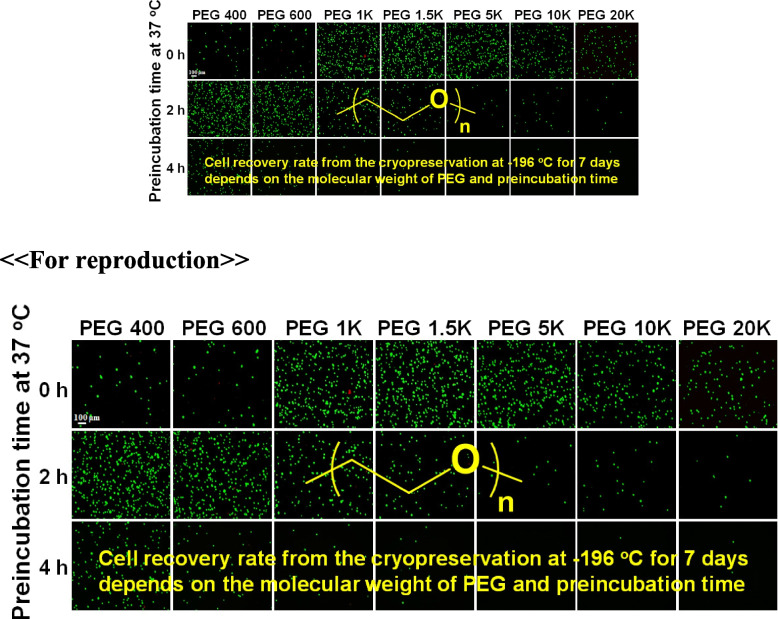

**Supplementary Information:**

The online version contains supplementary material available at 10.1186/s40824-023-00356-z.

## Background

Cells and stem cells are considered to be next generation medicine to innovate the current medical procedure [1]. However, there are many hurdles to overcome for the success of cell-based therapy. Cell cryopreservation is one of the critical issues. Ice (re)crystallization can occur inside and outside of cells during cryopreservation of the cells. Both ice crystallization (during cooling) and recrystallization (during melting) induce not only chemical damage to the cells by osmotic pressure development but also physical damage to the cells by ice crystals [2, 3]. During the cryopreservation procedure, the unfrozen part becomes concentrated with solutes in the medium, unbalancing the cells’ osmotic pressure, followed by the dehydration or swelling of cells. To avoid the damage caused by osmotic pressure development, the current state-of-the-art method is to use a medium containing dimethyl sulfoxide (DMSO) (10% v/v). DMSO freely diffuses into the cell, dehydrates the cell, and replaces the intracellular water. Thus, it reduces the osmotic pressure development in the cells during cryopreservation [4, 5]. However, DMSO is cytotoxic, and most cells die when exposed to DMSO 10% at 37 °C, furthermore, DMSO can be involved in post-differentiation mechanisms of the stem cells [6]. A series of synthetic polymers such as poly(vinyl alcohol), carboxyated ε-poly(L-lysine), poly(ethylene glycol)-poly(L-alanine), polybetaine, and trehalose-grafting polystyrene have been developed to replace DMSO as a cryoprotectant of cells [4, 6–9]. However, new compounds are required to prove biocompatibility. For example, poly(L-alanine-co-L-lysine) shows excellent IRI activity comparable with poly(vinyl alcohol), however, it suffers from the cytotoxicity [10]. As for biocompatible polymers, poly(ethylene glycol)s (PEGs) are the most important candidates. PEGs are widely used for drug formulation and modification due to their proven biocompatibility for human uses as parenteral excipients as well as modification (PEGylation) of protein drugs and anticancer drugs [11, 12]. The dynamic nature of PEGs in water not only prevents the approach of proteolytic enzymes but also decreases glomerular filtration rate, lengthening the plasma half-life of the PEGylated drugs. The hydrophilic nature of PEGs contributes to the improved solubility of PEGylated drugs. Recently, antibodies to PEG were reported [13]. Hence, the new PEGylated drugs are required to prove their immunosafety standards of the United States Food and Drug Administration (FDA). If PEGs are conjugated to macromolecules, they become immunogenic, similar to haptens which become immunogenic only when conjugated with large molecules [14, 15]. However, free PEG stands its position as the most biocompatible among the synthetic polymers and has also recently been used in the lipid nanoparticle (LNP) preparation of the corona vaccine [16].

Here, we report that PEGs can be used as an important cryoprotecting agents for mesenchymal stem cells (MSCs). Depending on the molecular weight, cell permeability or location of PEGs, and thus their working mechanisms might vary. For example, intracellular PEG might contribute to the suppression of osmotic pressure as well as the inhibition of ice recrystallization inside cells. To investigate these effects for the cryopreservation of MSCs, the cells were preincubated for 0 h (no incubation), 2 h, and 4 h at 37 °C in the presence of PEGs (10% in medium) before cryopreservation. The molecular weight of the PEGs varied over 400, 600, 1000 (1 K), 1500 (1.5 K), 5000 (5 K), 10,000 (10 K), and 20,000 (20 K) Da. In addition, the cryopreservation mechanism of PEGs, depending on their molecular weights, was studied through IRI, INI, membrane stabilization of cells, and cell internalization of PEGs. Finally, the recovered MSCs from cryopreservation at –196 °C for 7 days were studied for proliferation and osteo-, chondro-, and adipogenic differentiation to prove their healthy states.

## Methods

### Materials

PEGs (400 Da: TCI, Korea; 600 & 10 K: Sigma Aldrich, USA, 1 K & 1.5 K: Alfa Aesar, USA, 5 K & 20 K: Fluka, Switzerland) were used as received. Cell culture media and supplements like fetal bovine serum (FBS), penicillin, and streptomycin were purchased from Corning, USA.

### Cryopreservation and cell recovery

The PEG solutions were prepared in Dulbecco’s Modified Eagle Medium (DMEM) by dissolving each PEG with the molecular weight of 400, 600, 1 K, 1.5 K, 5 K, 10 K, and 20 K Da at 10 wt.% concentration. Tonsil tissue-derived MSCs were donated by the Ewha Womans University Medical School (Seoul, Korea). The cells were cultured with DMEM supplemented with FBS (10% v/v), antibiotic-antimitotic (1% v/v), and penicillin/streptomycin (1% v/v) at 37 °C in a sterile incubator under 5% CO_2_ atmosphere to obtain passage 6 cells. The confluent TMSCs were collected using trypsin/ethylenediamine tetraacetic acid solution. The cells were added at a density of 1.0 × 10^6^ cells to 1 ml medium containing 10% of PEG in the cryovials (Thermo Scientific Nunc, USA), which were incubated at 37^o^ C under 5% CO_2_ conditions for different time periods of 0 h (no incubation), 2 h, and 4 h. Then, the cryovials were cooled to –80 °C in a cryobox at a cooling rate of –1^o^ C/min. After 12 h incubation at –80 oC, the cryovials were transferred to liquid nitrogen (–196 °C) and cryopreserved for 7 days. A 10% DMSO in DMEM (DMSO 10%) was used as a control.

After 7 days of cryopreservation at –196 °C, the cryovials were removed from the liquid nitrogen and thawed in a 37 °C water bath. The cell suspension was transferred into a conical tube and tenfold diluted with the growth media. Then, the cells were collected by centrifugation (1500 rpm) for 5 min. The supernatant was discarded, and the cell pellet was resuspended in 500 μl of growth media. Afterward, the cell suspension was evaluated for immediate cell viability using a live/dead assay kit (Invitrogen, CA, USA). In addition, the cells were transferred to individual wells of a 24-well plate and cultured for 24 h in a humidified 5% CO_2_ atmosphere at 37 °C. Then, the cells were collected using trypsin treatments, followed by washing with phosphate buffered saline (PBS). After centrifugation, the cells were resuspended in media and evaluated with trypan blue and a live/dead assay kit for cell recovery assessment [6].

### Responses of adhered cells and suspended cells to PEG solutions

MSCs were seeded in a 24-well plate (5.0 × 10^4^ cells/well), and the adhered cells were exposed to the culture medium containing PEGs (10 wt.%) to evaluate their toxicity. After 24 h of exposure, cell viability was tested by a live/dead kit and a cell counting kit-8 (CCK-8, Dojindo, Japan). Cells treated with DMSO 10% and growth media were studied as negative and positive controls, respectively. In addition, the cytotoxicity of PEG 10 wt.% solution was evaluated for the suspended cells. The cells suspended in media containing PEG (10 wt.%) were incubated for 2 h at 37 °C. The cell viability was analyzed with the live/dead kit and CCK-8.

### Ice recrystallization inhibition (IRI)

The DMEM containing PEG (10 wt.%) was studied for IRI activity by the splat assay method [17, 18]. A 10 μl droplet of media containing PEG (10 wt.%) was dropped from 1.4 m onto a microscope slide on top of an aluminum foil cooled to –78^o^ C using dry ice. The droplet immediately froze and formed a thin wafer of ice. Then, the slide glass was placed in a cryostage at –6 °C, and annealed for 30 min. Cryo-microscopy images of the wafer were then taken through crossed polarizers using a microscope (CX40IT, Soptop, China) equipped with a 10 × /0.25/∞/-/FN26.5 lens (UIS-2, Olympus Ltd., Japan) and 3 M CMOS color digital camera (BoliOptics, USA). Photographs were taken from three different locations, and the average crystal size was calculated by using ImageJ. DMEM without PEGs was used as a control for IRI activity and assigned to be 100% in the mean largest grain size (MLGS) analysis.

### Ice nucleation inhibition (INI)

Aqueous PEG polymer solutions (10 wt.%) were filtered through a poly(vinylidene fluoride) filter (0.22 μm, Whatman, Fisher Scientific, USA). A total of 20 droplets (0.5 μl/droplet) for each PEG solution were pipetted onto a slide glass and placed inside the cryostage (LTS120, Linkam Scientific Instruments Ltd, UK). The cryostage was rapidly cooled to 5 °C at a rate of 30 °C/min and kept it for 3 min. The sample was then cooled to –30 °C at a cooling rate of –1 °C/min. The frozen fraction of the droplets was monitored. The nucleation temperatures defined by the temperatures at which half of the droplets were frozen were recorded [19].

### Membrane stabilization

Cell membrane stabilization was studied by the published method [20]. First, MSCs were suspended and incubated with 1.0 μM calcein AM (BD Bioscience, UK) for 30 min in PBS at 37 °C. The cells were then centrifuged and rinsed with PBS to remove the calcein AM outside of the cells. Then, the cells were resuspended in DMEM (2.0 ml). The cell suspension about 50 μl were then placed to prepare a density of 2.0 × 10^5^ cells/well in 96-U-well plates and centrifuged at 500 rpm for 2 min to sink down the cells. Afterward, the PEG solution (20.0 wt.%, 100 μl) and trypan blue (0.32%, 50 μl) were added to obtain a final concentration of 10 wt.% (PEG) and 0.08% (tryphan blue), respectively. The plate was then analyzed in a fluorescence plate reader (Spectramax i3, Molecular Devices, USA) at every 10 min under 485(excitation)/530(emission) nm for 180 min at 37 °C. Cells treated with DMSO (10% or 20%) in DMEM and DMEM itself (control) without both PEG and DMSO were assayed for comparison.

### Mechanism of PEG internalization into the cells

To study the internalization mechanism, MSCs were cultured in 100 pi dishes (diameter = 100 mm, 7.0 × 10^5^ cells/well) for 24 h. Then, the cells were separately treated without (control) or with inhibitors of macropinocytosis (rottlerin, 5.0 μg/ml), caveolae-mediated endocytosis (filipin, 5.0 μg/ml), and clathrin-mediated endocytosis (chlorpromazine, 15.0 μg/ml) for 30 min [21–23]. Subsequently, the cells were incubated with PEG 600, PEG 1.5 K, and PEG 20 K at 100 μM for another 2 h at 37 °C in starvation media. Then, adhered cells on the culture plate were washed with PBS (3 times) and collected using trypsin. Cells were resuspended in PBS (500 μl) and ultrasonicated (Ultrasonic Processor, Sonics and Materials Inc. USA) (30amp, 1 min). Next, cells were centrifuged at 9000 g for 20 min to remove cellular debris and collect the supernatant. The PEG in the supernatant was quantified using enzyme-linked immunosorbent assay (ELISA) kits of PEG (MyBioSource, USA).

### Proliferation of recovered cells

The proliferation of recovered MSCs from PEG 400*, PEG 600*, PEG 1 K, PEG 1.5 K, and PEG 5 K solutions was assayed on days 1, 3, and 5 using CCK-8. PEG 400* and PEG 600* indicate that cells were preincubated for 2 h at 37 °C before cryopreservation. The recovered live cells (5.0 × 10^4^ cells/well) were seeded to the 24-well plate with the growth media. The proliferation of MSCs recovered from cryopreservation using the traditional DMSO 10% system was also compared as a control. The cell images were obtained at 1, 3, and 5 days during the cell proliferation using the live/dead assay kit. Briefly, the cells were treated for 30 min with ethidium homodimer-1 (2.0 μM) and calcein AM (2.0 μM) solution. The fluorescence images were obtained using an Olympus IX71 fluorescence microscope and Olympus DP2-BSW software. At 1, 3, and 5 days of proliferation, the growth medium was replaced with CCK-8 solution (10% in DMEM containing 1.0% PS), and incubated for 150 min at 37 °C. The absorbance was measured at 450 nm relative to 655 nm using a microplate reader (iMARK, Bio-Rad, USA) for a quantitative assay for cell proliferation. 100% was assigned to 0 days (3 h) of data.

### Multipotency of recovered MSCs for differentiation

The multilineage differentiation potentials of cryopreserved MSCs were evaluated for osteo-, chondro-, and adipogenic differentiation. The cells were seeded in 24-well plate (5.0 × 10^4^ cells/well). After confluence, the cells were treated with specific osteo-, chondro-, and adipogenic induction media [24]. The differentiation media was replaced every 2 days with fresh media for two weeks. On the last day of differentiation, the cells were fixed with 4% formaldehyde and washed with PBS. Thereafter, the fixed cells were stained with alizarin red, alcian blue, and oil red O for osteo-, chondro-, and adipogenic differentiation, respectively. The images were captured by an Olympus IX71 fluorescence microscope using Olympus DP2-BSW software.

### Statistical analysis

All the experiments were carried out in triplicates for statistical treatments. The significance of the values was analyzed using one-way ANOVA with Tukey tests. Differences were considered statistically significant when the p-value was less than 0.05 and 0.01, marked as * and **, respectively.

## Results

**Fig. 1:**
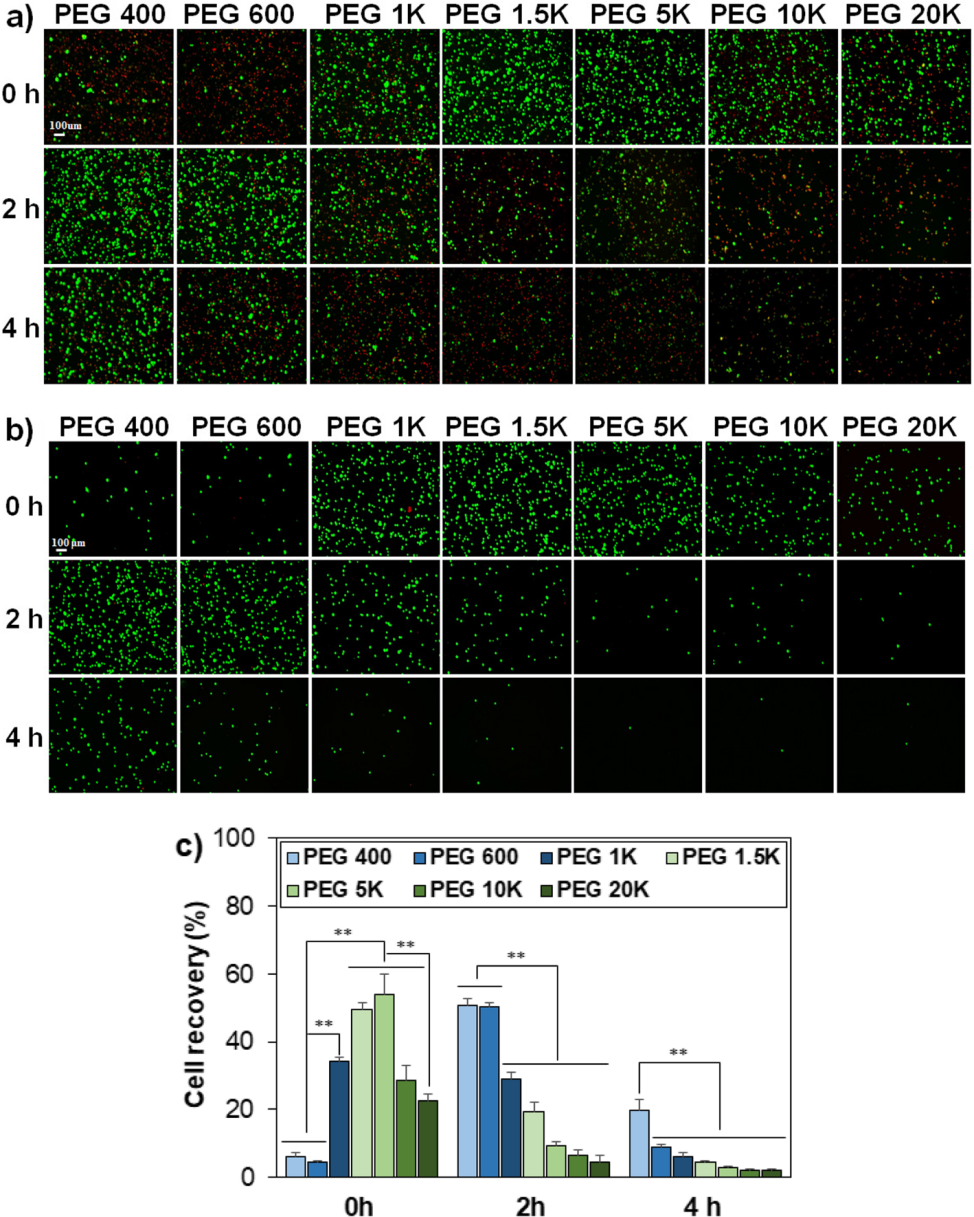
Fluorescence images of MSCs recovered from cryopreservation at –196 °C for 7 days, without post-thaw culture (**a**) and with 24 h post-thaw culture at 37 °C (**b**). The scale bar is 100 µm. PEGs as cryoprotectants varied their molecular weight over 400, 600, 1 K, 1.5 K, 5 K, 10 K, and 20 K Da at a concentration 10 wt.%. 0 h, 2 h, and 4 h indicate preincubation time of MSCs in the presence of PEGs at 37 °C before cryopreservation. **c**) Quantitative analysis of cell recovery for the post-thaw culture systems at 37 °C for 24 h. ** indicates *p* < 0.01. *N* = 3

Cell permeability and thus the location of PEGs, whether they are inside or outside of the cells, depends on the molecular weight of PEGs. Facile diffusion of the PEGs into the cell might relieve the osmotic pressure development and inhibit ice crystallization inside the cell. Therefore, the molecular weight effect is expected to play a role in the cryopreservation of cells. Before the cryopreservation, MSCs were suspended in the medium containing PEG 10%, followed by preincubating at 37 °C for 0 h (no incubation), 2 h, and 4 h, where the molecular weights of PEGs varied over 400, 600, 1 K, 1.5 K, 5 K, 10 K, and 20 K Da. Then, the cells were subjected to cryopreservation at –196 °C for 7 days by slow-freezing protocol [25, 26]. The live/dead images of the recovered cells just after cryopreservation (Fig. 1a) and 24 h after post-thaw culture at 37 °C for the recovered cells (Fig. 1b) are shown. Dead cells stained in red were removed during post-incubation. 24 h post-thaw culture is usually applied in assaying the cell recovery because many cells are weak and die in several hours after the recovery from –196 °C [6, 27, 28]. Cell recovery was significantly affected by the molecular weight of PEGs and the preincubation time. As for the cells in a medium containing low molecular weight PEGs (PEG 400 and PEG 600), 2 h preincubation time was needed to achieve a significant cell recovery from cryopreservation. Meanwhile, the medium containing intermediate molecular weight PEGs (PEG 1 K, PEG 1.5 K, and PEG 5 K) reduced the cell.

Recovery by preincubation and excellent cell recovery was achieved without (0 h) preincubation. High molecular weight PEGs (PEG 10 K and PEG 20 K) were ineffective in cryoprotection of the cells with or without preincubation. CCK-8 was used to quantitatively analyze the cell recovery (Fig. 1c). The cell recovery using a traditional method of DMSO 10% in the medium was 70 ± 2.8% by the same protocol (Figure S1). However, there are toxicity concerns of residual DMSO. Only 25% of the MSCs survived when exposed to DMSO 10% at 37 °C for 24 h (Figure S2). The PEG 10% in the medium used in the current study has advantages in this regard. Without preincubation (0 h incubation) at 37 °C, the cell recovery was less than 10% when PEG 400 or PEG 600 were used as a cryoprotectant. However, cell recovery significantly improved to 34%, 50%, and 54% when PEG 1 K, PEG 1.5 K, and PEG 5 K were used as cryoprotectants, respectively. On the other hand, cell recovery significantly improved from < 10% to 51% by preincubating the cells in a medium containing PEG 400 or PEG 600 at 37 °C for 2 h. The cell recovery gradually decreased by the 2 h preincubation protocol as the molecular weight of PEG increased to 20 K Da. However, preincubating the cells at 37 °C for 4 h in the presence of PEGs with 400–20 K Da was detrimental to the cells, and the recovery decreased to < 20%. To conclude, the cell recovery was strongly affected by the molecular weight of PEGs and preincubation time.

To understand the cell recovery behavior depending on the molecular weight of PEGs and incubation time, we investigated several scenarios. First, cell death might occur during preincubation at 37 °C for 2 h because the MSCs are in a suspended state during preincubation in the presence of PEGs 10 wt.%. To verify this, the cell viability was compared for the cells adhered to the culture plate and the cells suspended in the solutions in the presence of PEG 10 wt.%. The images of adhered MSCs reflected the total cells and exhibited typical stretched cell shapes. A part of the suspended cells was sampled and images of the cells exhibited cell aggregation in the PEG solutions as the molecular weight of PEG is higher than 1.5 K Da. Many cells were dead in the solutions.

**Fig. 2:**
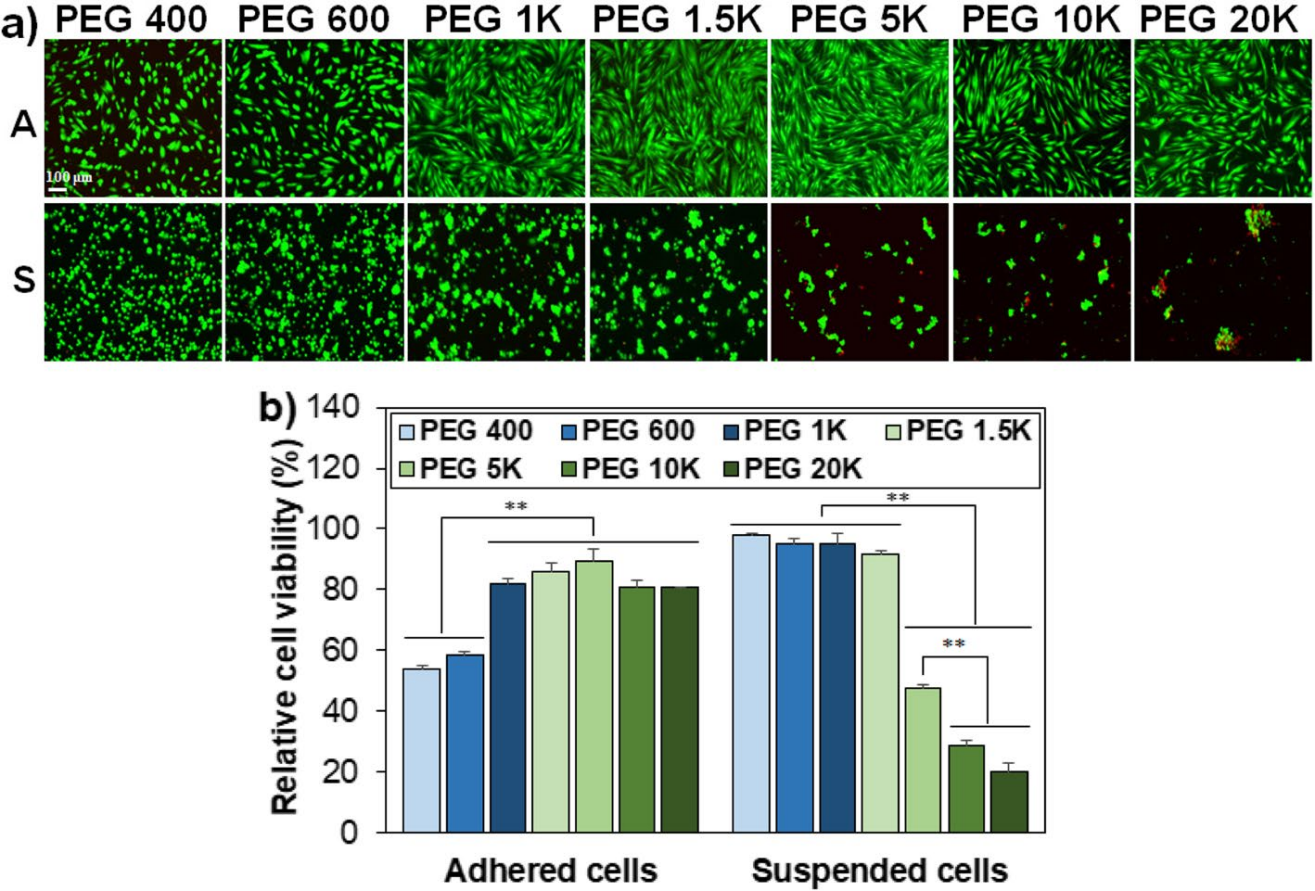
**a** Live/dead images of PEG-treated cells adhered to the culture plate (A) and suspended in the medium (S). The scale bar is 100 μm. **b** Quantitative assay of cell viability for adhered cells and suspended cells. The relative cell viability in DMEM in the presence of PEGs compared with in the absence of PEGs (100%). Cell viability was assayed using the CCK-8. ** indicates *p* < 0.01, *N* = 3

Containing PEG molecular weight > 5 K Da (Fig. 2a). Quantitative analysis of the cell viability using CCK-8 indicated that PEG 400 and PEG 600 are rather toxic and exhibited < 60% cell viability for the adhered cells. The cell viability was 80%–90% for adhered cells in PEG 1 K–20 K solutions. In contrast, PEG 400–1.5 K Da are cytocompatible with the suspended cells. However, > 50% cell death was observed for the suspended cells in solutions containing PEGs > 5 K Da (Fig. 2b). The data proves that the relatively low cell recovery of the systems containing PEG 5 K–20 K Da after preincubation of cells at 37 °C for 2 h and 4 h is related to cell death during preincubation before the cryopreservation.

**Fig. 3:**
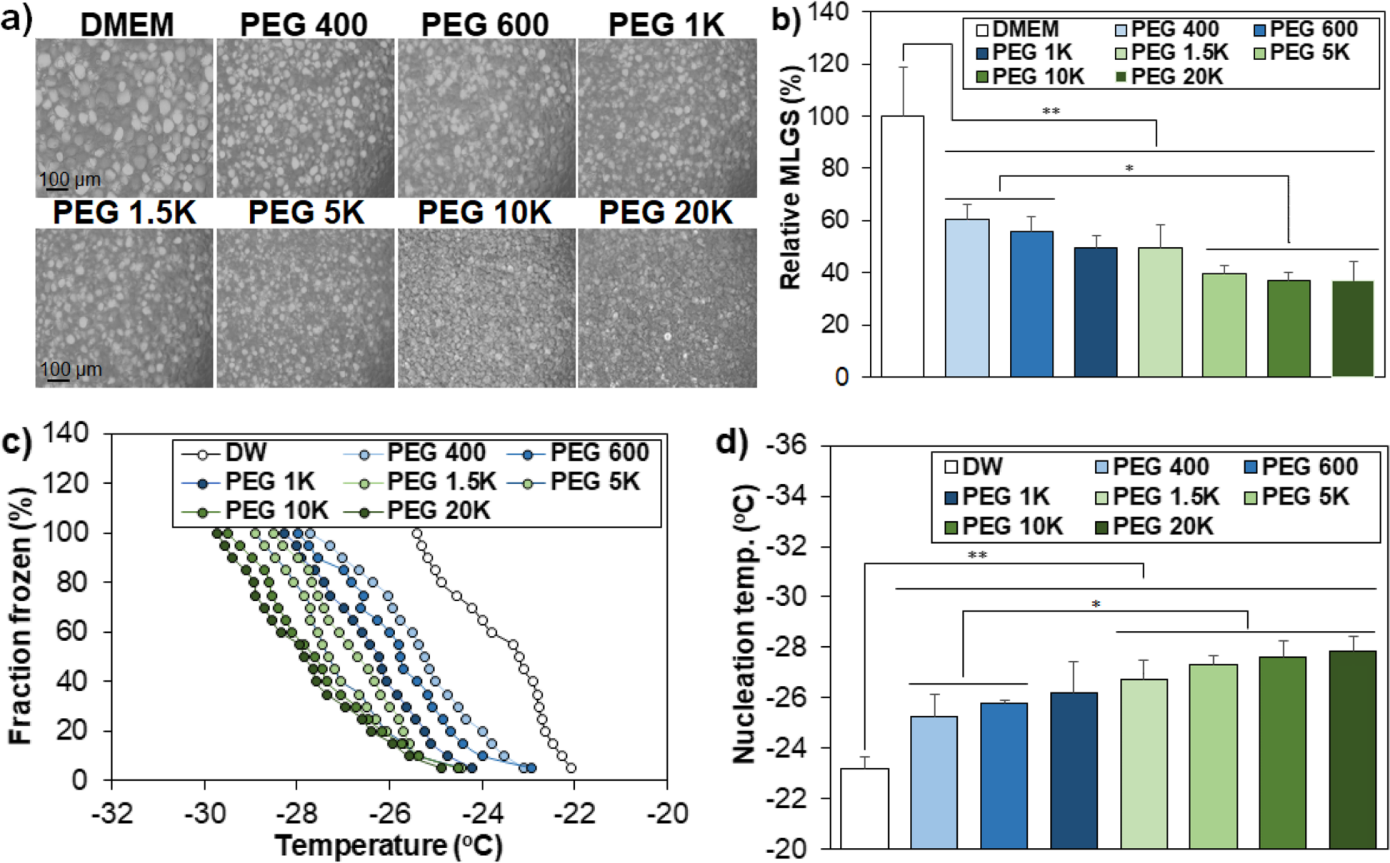
Inhibition of growth and nucleation of ice crystals. **a** Microscopy images of ice crystals in the presence of PEGs solutions (10 wt.%) in DMEM. The scale bar is 100 μm. **b** MLGS of PEG solutions relative to that of DMEM without PEGs. c) Fraction frozen of the droplets as a measure of INI activity of aqueous PEG solution (10 wt.%). d) Nucleation temperature at which 50% of the 20 droplets was frozen. * and ** indicate *p* < 0.05 and *p* < 0.01, respectively. *N* = 3

Second, IRI activity is a well-known cell protection mechanism of cryoprotectants during cryopreservation [4, 6]. IRI activity was investigated in medium (DMEM) containing PEG 10%, and the ice crystal images were compared (Fig. 3a). The ice crystal size was significantly reduced in PEG 10% solutions compared with the control solution without PEG. The mean largest grain size (MLGS) of the PEG 400 system reduced to 60% of that of the control. MLGS further decreased to 39% − 37% of the control as the molecular weight of PEGs increased to 5 K − 20 K Da (Fig. 3b). In addition, the INI activity of PEGs exhibited a similar trend to the IRI activity, indicating that PEGs inhibit nucleation as well as recrystallization of ice crystals. Fraction frozen significantly decreased in the PEG solutions compared to the control system without PEG (Fig. 3c). This trend was analyzed by comparing the nucleation temperature at which half of droplets is frozen (Fig. 3d). The nucleation temperature steadily decreased from –23 °C (control without PEG) to –25 °C, (PEG 400) and to –28 °C (PEG 20 K) as the molecular weight of PEG increases (Fig. 3d).

**Fig. 4:**
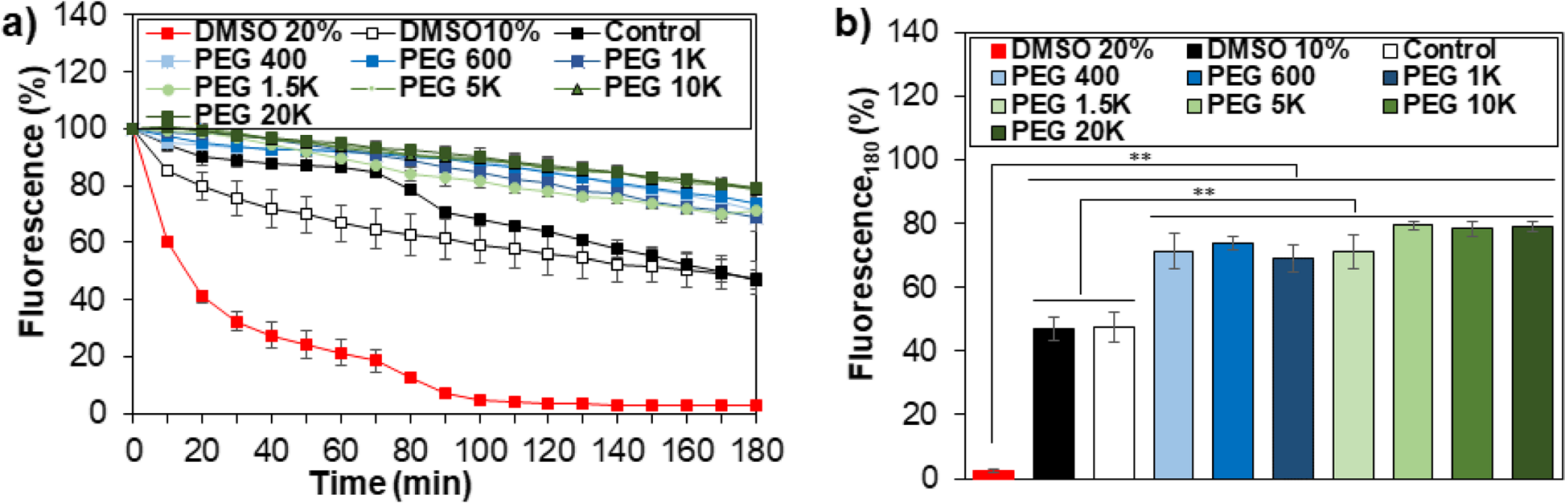
Membrane stabilization of MSCs by PEGs. **a** Changes in fluorescence intensity in the medium containing PEGs. Control is the medium containing neither DMSO nor PEGs. DMSO 20% and DMSO 10% indicate the medium containing and 20% and 10% of DMSO, respectively. **b** Comparison of the fluorescence intensity at 180 min from plot a). **: *p* < 0.01. *N* = 3

Third, membrane stabilization was studied by fluorescence decay of calcein AM [20]. Nonfluorescent calcein AM enters the live cell and emits green fluorescence after hydrolysis of the acetoxymethyl ester bond to calcein by esterases in the cytoplasm. Trypan blue enters the cell with destabilized membrane and quenches the fluorescent intensity of calcein in the cytoplasm. As the membrane is destabilized, thus, the fluorescent intensity decreases. DMSO 20% solution is very effective in destabilizing the membrane, as shown by the fast decay in the fluorescent intensity (Fig. 4a). Compared with the control (without PEG) and DMSO 10%, PEG 10% solutions stabilized the cell membrane, as shown by a significantly slower decay rate of the fluorescent intensity (Fig. 4b). However, there was no statistical difference among the PEG solutions containing different molecular weights over 400 − 20 K Da. Therefore, the cell membrane is significantly stabilized in the PEG solutions (10 wt.%) regardless of the PEG molecular weight in a range of 400 − 20 K Da.

**Fig. 5:**
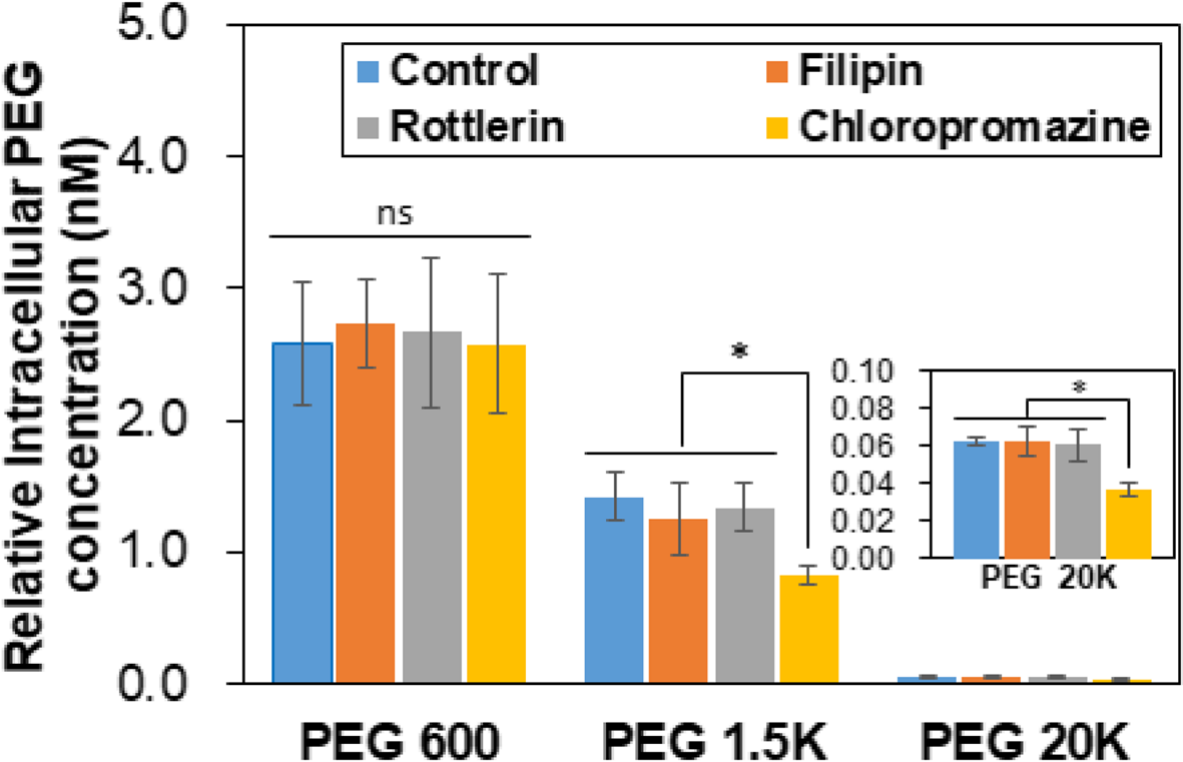
Internalization of PEGs into the MSCs in the presence of inhibitors. PEG 600, PEG 1.5 K, and PEG 20 K were used. Control is the data in the absence of an inhibitor. ns: nonsignificant. *: *p* < 0.05. *N* = 3

Fourth, the permeability of PEGs into the cell was investigated by assaying intracellular PEGs using a PEG ELISA kit. PEGs with a molecular weight of < 1 K Da easily permeate the plasma membrane of most cells [23, 29]. The cell penetration of PEG 5 K with a fluorescent tag of rhodamine B was internalized into L929 (fibroblast) cells, Hela cells, and Michigan Cancer Foundation-7 (MCF-7) cells by micropinocytosis [23]. However, the rhodamine B tag might affect the internalization procedure of PEG into cells. Hence, internalization of pristine PEGs was investigated for A549 Madin-Darby canine kidney (MDCK)-mock cells (non-small cell lung cancer cells) using liquid chromatography interfaced with a mass spectrophotometer [29]. They reported that PEGs < 2 K Da were internalized by passive diffusion, and PEG > 5 K Da internalized into the A549 MDCK-mock cells by caveolae-mediated endocytosis. We assayed the internalization of pristine PEGs into the MSCs using the ELISA kit. PEG 600 (low molecular weight), PEG 1.5 K (intermediate molecular weight), and PEG 20 K (high molecular weight) were selected, and PEG solutions without inhibitors (control) and with the inhibitors rottlerin, filipin, and chlorpromazine were compared. Rottlerin, filipin, and chlorpromazine are inhibitors of macropinocytosis, caveolae-mediated endocytosis, and clathrin-mediated endocytosis, respectively, [21–23]. There was no difference among the systems with and without (control) inhibitors for PEG 600, indicating that PEG 600 enters the cells by passive diffusion (Fig. 5). On the other hand, internalization of PEG 1.5 K into the MSCs reduced to about half of PEG 600, and was further suppressed to 32% of that of PEG 600 by chlopromazine treatments. This indicates that clathrin-mediated endocytosis as well as reduced diffusion is involved in the internalization of PEG 1.5 K. In addition, PEG 20 K exhibited a significant decrease in the internalization to about 1% − 2% of PEG 600. Similar to PEG 1.5 K, the chlopromazine treatments reduced the internalization, indicating that the major mechanism of PEG 20 K internalization into MSCs also is clathrin-mediated endocytosis.

**Fig. 6:**
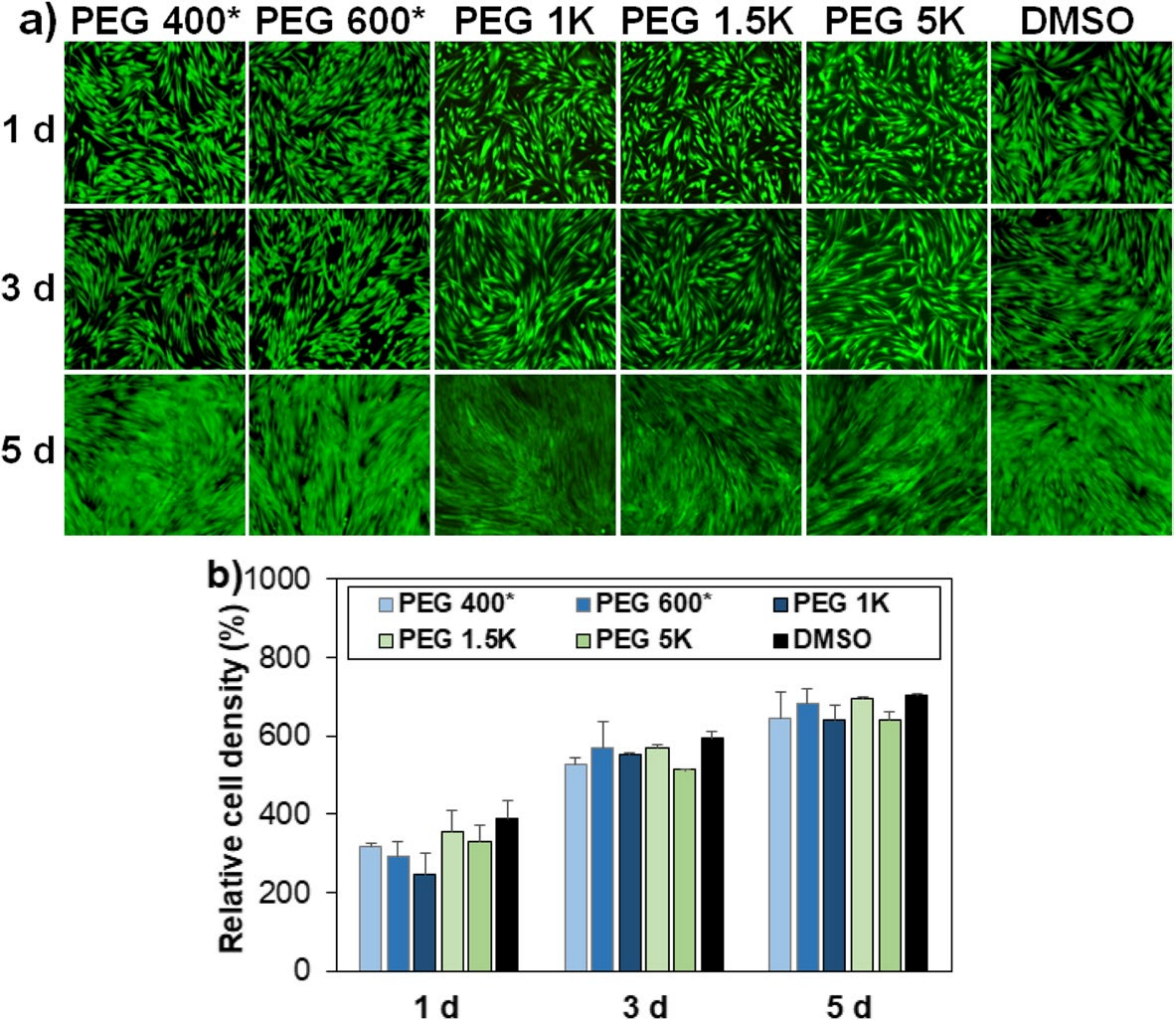
**a** Fluorescence images exhibiting proliferation of MSCs recovered from cryopreservation at –196 °C for 7 days. PEG 400* and PEG 600* indicate that the recovered cells had been preincubated for 2 h at 37 °C before cryopreservation. DMSO indicates the traditional system using DMSO 10% for cryopreservation of MSCs. The scale bar is 100 μm. **b** Quantitative analysis of cell proliferation relative to day 0 (100%) assayed by the CCK-8

The MSCs recovered from cryopreservation at –196 °C for 7 days were investigated for proliferation and differentiation. For the PEG 400 and PEG 600 systems, the cells selected were preincubated ones at 37 °C for 2 h before cryopreservation, and indicated as PEG 400* and PEG 600*, respectively. No preincubation was performed before cryopreservation for the PEG 1 K, PEG 1.5 K, and PEG 5 K systems because they provide enough cells due to their excellent cell recovery from the cryopreservation. The recovered cells from these systems exhibited excellent proliferation over 5 days, and 1.8–2.6-fold increases in cell number over 5 days (Fig. 6a and 6b).

**Fig. 7:**
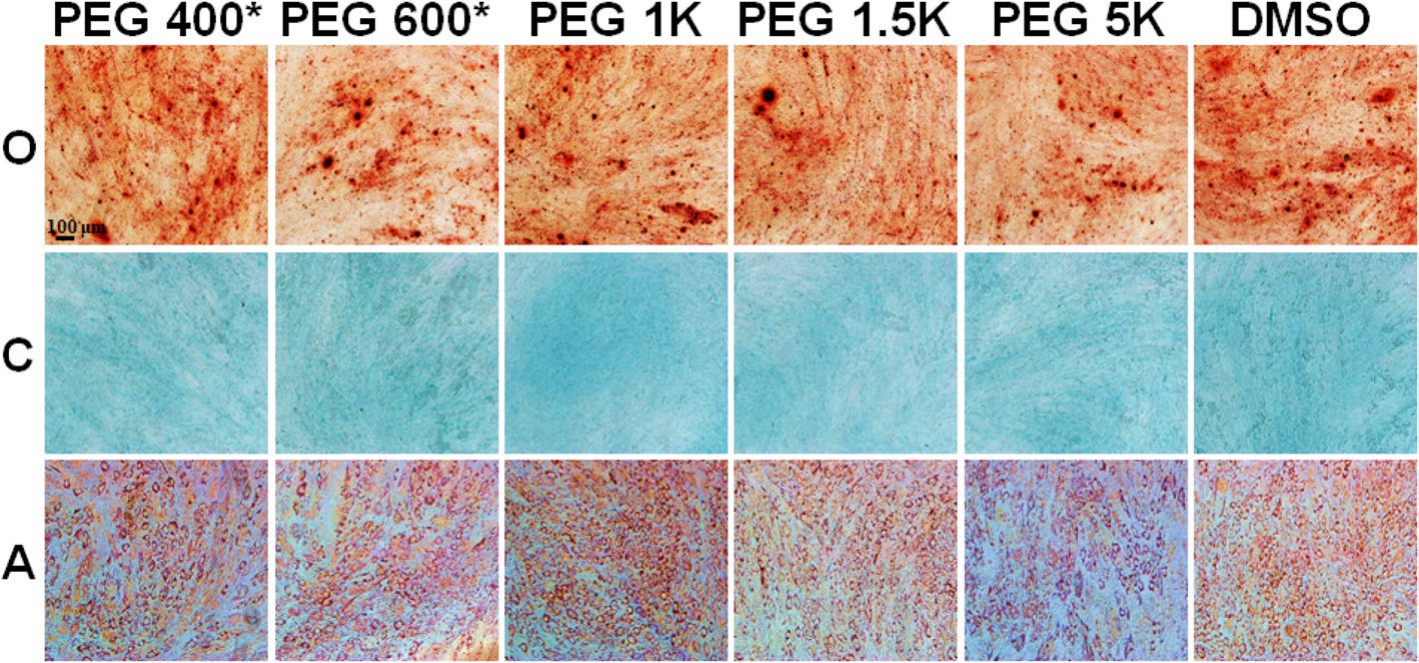
Differentiation of recovered MSCs into osteocytes, chondrocytes and adipocytes. The cells were stained by alizarin red (osteogenic; O), alcian blue (chondrogenic; C), and oil red O (adipogenic; A) staining, respectively. The scale bar is 100 μm

In addition, their osteo-, chondro-, and adipogenic differentiation potentials were confirmed by staining alizarin red, alcian blue, and oil red O, respectively (Fig. 7). They stain calcium ions into brown, sulfated proteoglycan into blue, and lipids/triglycerides into red, respectively, indicating that osteo-, chondro-, and adipogenic differentiation of the MSCs, respectively [24]. All the cells recovered from cryopreservation exhibited excellent proliferation and differentiation, suggesting that they remain in healthy conditions.

## Discussion

PEGs, well-known biocompatible synthetic polymers, can be good cryoprotectants for MSCs. However, the cell cryopreservation protocol should differ depending on the molecular weight of PEGs. In this study, PEGs with various molecular weights (400, 600, 1 K, 1.5 K, 5 K, 10 K, and 20 K Da) were studied as a cryoprotectant of MSCs by varying the preincubation time of 0 h (no incubation), 2 h, and 4 h at 37 °C in the presence of various PEGs at 10 wt.% before the cryopreservation. The cell recovery was excellent only after 2 h preincubation for PEG 400 and PEG 600, whereas it was good without preincubation for PEG 1 K, PEG 1.5 K and PEG 5 K. To understand why cryoprotection capability is different depending on PEG molecular weight and incubation time, various studies such as IRI, INI, membrane stabilization, and intracellular transport of PEGs were performed. Low molecular weight PEGs (400 and 600 Da) are compatible for suspended cells during incubation at 37 °C for 2 h, and they have high permeability to cells by simple diffusion. Thus, preincubation in the presence of low molecular weight PEGs before cryopreservation could improve cryoprotection. The internalized PEGs during preincubation might suppress osmotic pressure development during cryopreservation. Intermediate molecular weight PEGs (1 K − 5 K Da) contribute to cell recovery by IRI and INI mechanisms as well as membrane stabilization effects of the PEGs. Internalized PEG also marginally contributes to cell recovery. Therefore, these processes do not need internalization of the PEG, and thus, they do not need 2 h preincubation of the cells at 37 °C. In contrast, high molecular weight PEGs (10 K and 20 K Da) exhibited toxicity for suspended cells during preincubation at 37 °C for 2 h and they have a low permeability to cells. The high molecular weight PEGs were ineffective as a cryoprotectant.

## Conclusions

Low molecular weight PEGs (400 and 600 Da) exhibited cryoprotection of MSCs through internalized PEGs by diffusion which requires a 2 h preincubation time before cryopreservation. Intermediate molecular weight PEGs (1 K–5 K Da) work as cryoprotectants mostly by extracellular PEG through IRI, INI, and membrane stabilization, as well as partly by internalized ones by clathrin-mediated endocytosis. High molecular weight PEGs (> 10 K Da) kill the cells during preincubation and fail to work as a cryoprotectant. The recovered cells proliferate and undergo osteo-/chondro-/adipogenic differentiation, similar to the MSCs recovered from the traditional DMSO 10% system.

To conclude, PEGs can be used as a cryoprotectant. However, the detailed procedures, including preincubation, should consider the effect of the molecular weight of PEGs because the protection mechanism of IRI, INI, membrane stability, and cell permeability depend on PEG molecular weight.

## Supplementary Information

**Additional file 1**: **Figure S1.** Images of recovered cells form cryopreservation at -196 ^o^C for 7 days using DMSO 10% in DMEM. The cell recovery 24h after thawing was 70±2.8%. **Figure S2.** Live/dead images of cells adhered to the culture plate in DMEM and DMSO solution (10% in DMEM). The stem cells adhered to the culture plate were incubated at 37 ^o^C for 24 . The scale bar is 100 um. The cell viability assayed using the CCK-8 method was 25±0.4% and 100±0.98% in DMSO 10% and DMEM, respectively.

## Data Availability

For data requests, please contact the authors.

## Abbreviations

DMSO: Dimethyl sulfoxide
PEG: Poly(ethylene glycol)
MSC: Mesenchymal stem cell
IRI: Ice recrystallization inhibition
INI: Ice nucleation inhibition
Da: Dalton
FDA: Food and drug administration
LNP: Lipid nanoparticle
FBS: Fetal bovine serum
DMEM: Dulbecco’s modified eagle medium
PBS: Phosphate buffered saline
CCK-8: Cell counting kit-8
MLGS: Mean largest grain size
ELISA: Enzyme-linked immunosorbent assay
MCF-7: Michigan cancer foundation-7
MDCK: Madin-Darby canine kidney
