# Supplementary material for: Rediscovery of poly(ethylene glycol)s as a cryoprotectant for mesenchymal stem cells
Source: Biomater Res. 2023 Feb 20;27:17. doi: 10.1186/s40824-023-00356-z (PMC9942331; doi:10.1186/s40824-023-00356-z)

**Supplementary information**

Rediscovery of poly(ethylene glycol)s as a cryoprotectant for mesenchymal stem cells

Madhumita Patel, Jin Kyung Park, and Byeongmoon Jeong^*^

*Department of Chemistry and Nanoscience, Ewha Womans University, 52 Ewhayeodae-gil, Seodaemun-gu, Seoul, 03760, Korea*

* Corresponding author.

E-mail addresses: [bjeong@ewha.ac.kr](mailto:bjeong@ewha.ac.kr), Tel.: +82 2 3277 3411; Fax: +82 2 3277 3419

**Figure S1.** Images of recovered cells form cryopreservation at -196 ^o^C for 7 days using DMSO 10% in DMEM. The cell recovery 24h after thawing was 70±2.8%.


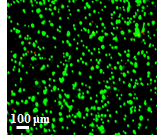


**Figure S2.** Live/dead images of cells adhered to the culture plate in DMEM and DMSO solution (10% in DMEM). The stem cells adhered to the culture plate were incubated at 37 ^o^C for 24 . The scale bar is 100 um. The cell viability assayed using the CCK-8 method was 25±0.4% and 100±0.98% in DMSO 10% and DMEM, respectively.


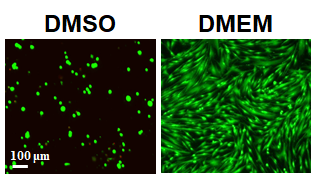

Supplement: Supplementary file 1 — Additional file 1: Figure S1. Images of recovered cells form cryopreservation at -196 oC for 7 days using DMSO 10% in DMEM. The cell recovery 24h after thawing was 70±2.8%. Figure S2. Live/dead images of cells adhered to the culture plate in DMEM and DMSO solution (10% in DMEM). The stem cells adhered to the culture plate were incubated at 37 oC for 24 . The scale bar is 100 um. The cell viability assayed using the CCK-8 method was 25±0.4% and 100±0.98% in DMSO 10% and DMEM, respectively. [file 40824_2023_356_MOESM1_ESM.doc]
